# BRAF and MEK Inhibitors Affect Dendritic-Cell Maturation and T-Cell Stimulation

**DOI:** 10.3390/ijms222111951

**Published:** 2021-11-04

**Authors:** Stefanie Hoyer, Valentina Eberlein, Gerold Schuler, Carola Berking, Lucie Heinzerling, Niels Schaft, Jan Dörrie

**Affiliations:** 1 Department of Dermatology, Universitätsklinikum Erlangen, Friedrich-Alexander-Universität Erlangen-Nürnberg (FAU), Hartmannstraße 14, 91052 Erlangen, Germany; stefanie.hoyer@uk-erlangen.de (S.H.); valentina.eberlein@izi.fraunhofer.de (V.E.); gerold.schuler@uk-erlangen.de (G.S.); carola.berking@uk-erlangen.de (C.B.); lucie.heinzerling@med.uni-muenchen.de (L.H.); 2 Comprehensive Cancer Center Erlangen European Metropolitan Area of Nuremberg (CCC ER-EMN), Östliche Stadtmauerstraße 30, 91054 Erlangen, Germany; 3 Deutsches Zentrum Immuntherapie (DZI), Ulmenweg 18, 91054 Erlangen, Germany; 4 Department of Palliative Medicine, Universitätsklinikum Erlangen, Comprehensive Cancer Center CCC Erlangen-EMN, Friedrich-Alexander-Universität Erlangen-Nürnberg (FAU), Krankenhausstraße 12, 91054 Erlangen, Germany; 5 Department of Genetics, Friedrich-Alexander-Universität Erlangen-Nürnberg, 91058 Erlangen, Germany; 6 Unit Preclinical Validation, Department of Vaccines and Infection Models, Fraunhofer Institute for Cell Therapy and Immunology, IZI, Perlickstraße 1, 40103 Leipzig, Germany; 7 Department of Dermatology and Allergy, University Hospital, LMU Munich, 80539 Munich, Germany

**Keywords:** vemurafenib, dabrafenib, cobimetinib, trametinib, BRAF inhibitor, MEK inhibitor, DCs, T cells, immunotherapy, melanoma

## Abstract

BRAF and MEK inhibitor (BRAFi/MEKi) combinations are currently the standard treatment for patients with BRAF^V600^ mutant metastatic melanoma. Since the RAS/RAF/MEK/ERK-pathway is crucial for the function of different immune cells, we postulated an effect on their function and thus interference with anti-tumor immunity. Therefore, we examined the influence of BRAFi/MEKi, either as single agent or in combination, on the maturation of monocyte-derived dendritic cells (moDCs) and their interaction with T cells. DCs matured in the presence of vemurafenib or vemurafenib/cobimetinib altered their cytokine secretion and surface marker expression profile. Upon the antigen-specific stimulation of CD8^+^ and CD4^+^ T cells with these DCs or with T2.A1 cells in the presence of BRAFi/MEKi, we detected a lower expression of activation markers on and a lower cytokine secretion by these T cells. However, treatment with any of the inhibitors alone or in combination did not change the avidity of CD8^+^ T cells in peptide titration assays with T2.A1 cells. T-helper cell/DC interaction is a bi-directional process that normally results in DC activation. Vemurafenib and vemurafenib/cobimetinib completely abolished the helper T-cell-mediated upregulation of CD70, CD80, and CD86 but not CD25 on the DCs. The combination of dabrafenib/trametinib affected DC maturation and activation as well as T-cell activation less than combined vemurafenib/cobimetinib did. Hence, for a potential combination with immunotherapy, our data indicate the superiority of dabrafenib/trametinib treatment.

## 1. Introduction

During the last decade, next generation sequencing allowed to uncover the mutational landscape of tumors. Melanoma was upon the first, for which this knowledge was translated into clinical applications. Virtually all cutaneous melanomas harbor driver mutations that activate the RAS/RAF/MEK/ERK MAPK pathway [1], which facilitates the proliferation and survival of the tumor cells [2,3,4,5]. More than half of all cutaneous melanomas express a mutated form of the BRAF oncogene, in which valine 600 is exchanged with glutamic acid (BRAF^V600E^; most commonly), lysine (BRAF^V600K^), or aspartic acid (BRAF^V600D^) [1], resulting in the constitutive activation of the pathway [6,7,8]. The second most frequent group of mutations activates NRAS, an upstream signaling protein [1,8,9].

BRAF^V600^ can be targeted with specific kinase inhibitors (BRAFi), i.e., vemurafenib (vemu; the first inhibitor approved by the FDA in 2011 as single treatment [10]), dabrafenib (dabra), or encorafenib, which have become a standard therapy in patients with this mutation [7,10,11,12]. Although the BRAFi are designed to target the mutated form, they show some paradox effects on wild type BRAF, resulting in an amplification of the signal [13]. To preempt the common relapses caused by resistant tumor variants [14], the BRAF^V600^ inhibitors were then combined with MEK inhibitors (MEKi), i.e., cobimetinib (cobi), trametinib (tram) [7], or binimetinib, which has been shown to increase progression-free survival significantly [13,15,16]. MEKi inhibit the non-mutated MEK, blocking the pathway in tumor and healthy cells alike. Three combinations of BRAFi/MEKi have been approved by the FDA and EMA for clinical use in the treatment of melanoma: (1) vemu + cobi (approved in November 2015 (FDA)/(EMA)), (2) dabra + tram (approved January 2014 (FDA)/July 2015 (EMA)), and (3) encorafenib + binimetinib (approved in June 2018 (FDA)/September 2018 (EMA)) [17]. Nevertheless, not all patients benefit from this treatment, and the majority suffers from disease recurrence after a median of 12–14 months [15,16]. Additional combination therapies are investigated, including combination with immunotherapy (reviewed in [18,19]), notably immune checkpoint inhibitors with moderate additional benefit [20,21,22]. Since different combination therapies are tested, it is important to understand how BRAFi/MEKi interact with immunotherapy to yield the best synergistic effects.

Therapeutic cancer vaccination is defined as the attempt to actively immunize a tumor-bearing individual against the tumor, thus inducing a host versus tumor reaction, which can support cancer therapy. Dendritic cells (DCs) are most commonly utilized as antigen-presenting cells, either by in vivo targeting [23,24] or by loading them with antigen ex vivo [25]. Since the RAS/RAF/MEK/ERK MAPK pathway is a key regulator pathway in different immune cells, the kinase inhibitors may also affect immune cells [26]. Other experimental approaches include bi-specific antibodies or the adoptive transfer of receptor-transfected T cells. For a combination of antigen-specific immunotherapy with targeted therapy, it is crucial to identify the most promising candidate that does not interfere with the immune function of DCs or T cells.

Therefore, we here intend to evaluate the effects of BRAFi/MEKi on the immunogenic key functions of DCs in an in vitro model studying DC/T-cell interactions. For that, we focused on the inhibitors dabra, tram, vemu, and cobi, and their combinations dabra/tram (D + T) and vemu/cobi (V + C). These agents have also recently been tested in clinical trials in combination with PD-1 checkpoint inhibition [20,22,27] as well as combinations with other immunotherapeutic agents (e.g., with a vaccine composed of six class II MHC-restricted helper peptides (NCT02382549), or with the oncolytic virus Talimogene Laherparepvec (NCT03088176)).

This study identified the critical effects of BRAFi/MEKi on DC function and T-cell stimulation, which should be considered in possible future combination therapies using BRAFi/MEKi and DC vaccination.

## 2. Results

### 2.1. Vemurafenib Treatment Affects Cytokine Secretion and Expression of Maturation Markers on MoDCs

To assess the effects of BRAF and MEK inhibitor (BRAFi/MEKi) treatment on moDC maturation, we applied vemurafenib (vemu, V), dabrafenib (dabra, D), trametinib (tram, T), and cobimetinib (cobi, C) either alone or in combination during the moDC maturation process (Table 1). The concentrations used in our experiments were chosen according to the plasma levels detected in patients treated with these BRAFi/MEKi (according to the instruction leaflets, prescription information, and publications) [28,29,30].

**Figure 1:**
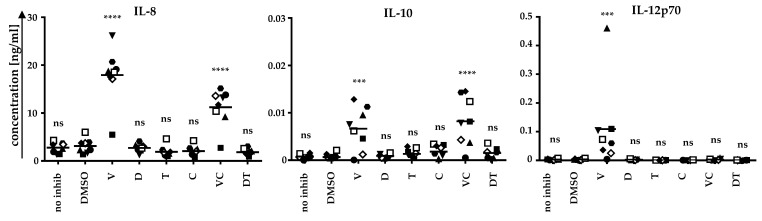
moDCs secrete IL-8, IL-10, and IL-12p70 upon treatment with vemurafenib during cytokine-induced maturation: moDCs were generated by plastic adherence, applying IL-4 and GM-CSF on days 1, 3, and 5. On day 6, cells were matured with IL-6, IL-1β, TNF, and PGE_2_. Cells were additionally treated without inhibitor (no inhib), with solvent control (DMSO), vemurafenib (V), dabrafenib (D), trametinib (T), cobimetinib (C), or the clinically used combinations V + C (VC) or D + T (DT). Supernatants were sampled after 24 h. Cytokine concentrations, analyzed by Cytometric Bead Array, of eight DC batches (represented by different symbols) of six donors (DC batches of the same donor, but generated in independent experiments, have the same symbol but a different color) assessed in independent experiments, are depicted. Bars indicate mean values. *p*-values were determined by one-way ANOVA. In the Dunnett’s multiple comparisons test, all conditions were tested against the solvent control DMSO. *** *p* ≤ 0.001, **** *p* ≤ 0.0001, ns: *p* > 0.05.

First, we analyzed whether BRAFi/MEKi application affects the cytokine secretion profile of the DCs (Figure 1). Therefore, moDCs were generated from the blood of healthy donors and were matured on day 6 with a cocktail consisting of IL-6, TNF, PGE_2_, and IL-1β [31]. Concomitantly, BRAFi (vemu or dabra) and MEKi (tram or cobi) were applied either alone or in combination. Untreated DCs and DCs treated with only the solvent control DMSO served as negative controls. After 24 h, supernatants were sampled, and cytokine secretion was analyzed by a Cytometric Bead Array (Figure 1).

Upon vemu treatment, IL-8, IL-12p70, and low quantities of IL-10 were secreted. The differences to the solvent control DMSO were highly significant. In combination with cobi, vemu also increased the secretion of IL-8 and IL-10. Neither dabra nor tram affected the cytokine secretion patterns of the DCs (Figure 1). Hence, vemu and V + C clearly change the cytokine secretion pattern of moDCs.

**Figure 2:**
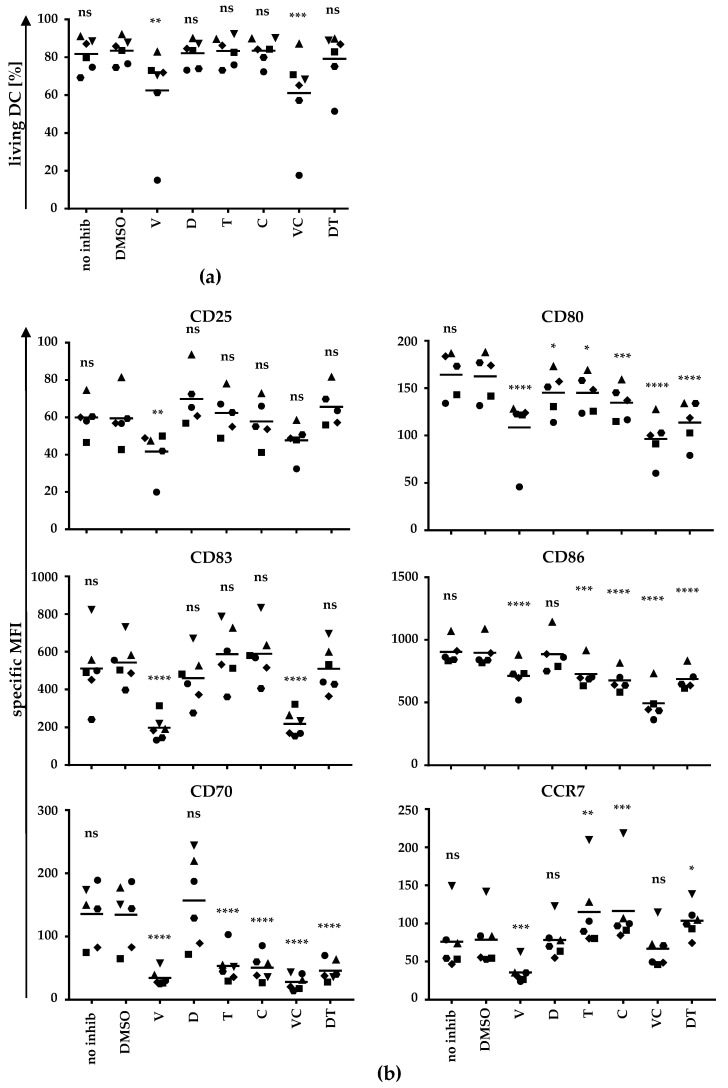
BRAF and MEK inhibitors partially inhibit DC maturation: moDCs were generated and treated as described in Figure 1. After 24 h, cells were harvested, stained for the indicated markers, and analyzed by flow cytometry. For the analyses of surface molecule expression of the indicated markers, cells were gated by forward (FSC) and side scatter (SSC). (**a**) The percentage of DCs in the life gate after treatment with different BRAF and/or MEK inhibitors or controls was determined. (**b**) The expression of surface markers is depicted as specific MFI (i.e., MFI after subtraction of background MFI of the respective isotype control antibodies). Data of six donors (represented by different symbols) assessed in independent experiments are shown. Bars indicate mean values. *p*-values were determined by one-way ANOVA. In Dunnett’s multiple comparisons test, all conditions were tested against the solvent control DMSO. * *p* ≤ 0.05, ** *p* ≤ 0.01, *** *p* ≤ 0.001, **** *p* ≤ 0.0001, ns: *p* > 0.05.

To address whether BRAFi and MEKi also affect DC maturation in terms of inducing changes in the maturation marker profile, we again generated moDCs and added BRAFi and MEKi alone or in combination during the maturation process. Cells were harvested after 24 h and were stained for the indicated maturation markers (Figure 2).

A life gate was defined by FSC/SSC to determine the fraction of living cells (Figure 2a). Vemu treatment significantly reduced the number of cells in the life gate. The combination of vemu and cobi also reduced the viability of the cells in a highly significant manner (Figure 2a). As described for effects on cytokine secretion, neither dabra nor tram affected the percentage of cells in the life gate. Thus, vemu and V + C not only influence cytokine secretion, but also affect moDC viability.

Analyzing the expression pattern of surface molecules after co-incubation with BRAFi/MEKi, we detected a significant reduction of CD25, CD80, CD83, CD86, CD70, and CCR7 expression by vemu treatment (Figure 2b). Dabra alone did not impair the expression of these maturation markers, except for CD80, which was significantly reduced, while CD25 and CD70 were slightly (although not significantly) increased (Figure 2b). The MEKi tram and cobi alone suppressed the upregulation of CD80, CD86, and especially of CD70 during moDC maturation, whereas CD25 and CD83 expression was unaffected (Figure 2b). Notably, CCR7 expression was significantly increased by tram or cobi treatment. The combination V + C also decreased the expression of the indicated markers similar to vemu alone, except for CD25 and CCR7, which were not affected. The observed weak effects of dabra on the expression profile of the indicated markers obviously had no influence on the inhibitory effects of tram on CD80, CD86, and CD70 expression (Figure 2b).

Even though tram and cobi alone had an impact on the expression of CD80, CD86, and CD70, they did not affect the expression of CD25 and CD83. In contrast, vemu and V + C not only changed the cytokine secretion pattern of the DCs, but also influenced viability and inhibited the upregulation of maturation markers during the moDC maturation process. Thus, the combination of V + C had a more negative impact on moDCs during their maturation process.

### 2.2. BRAF and MEK Inhibitors Do Not Affect T-Cell Avidity

**Figure 3:**
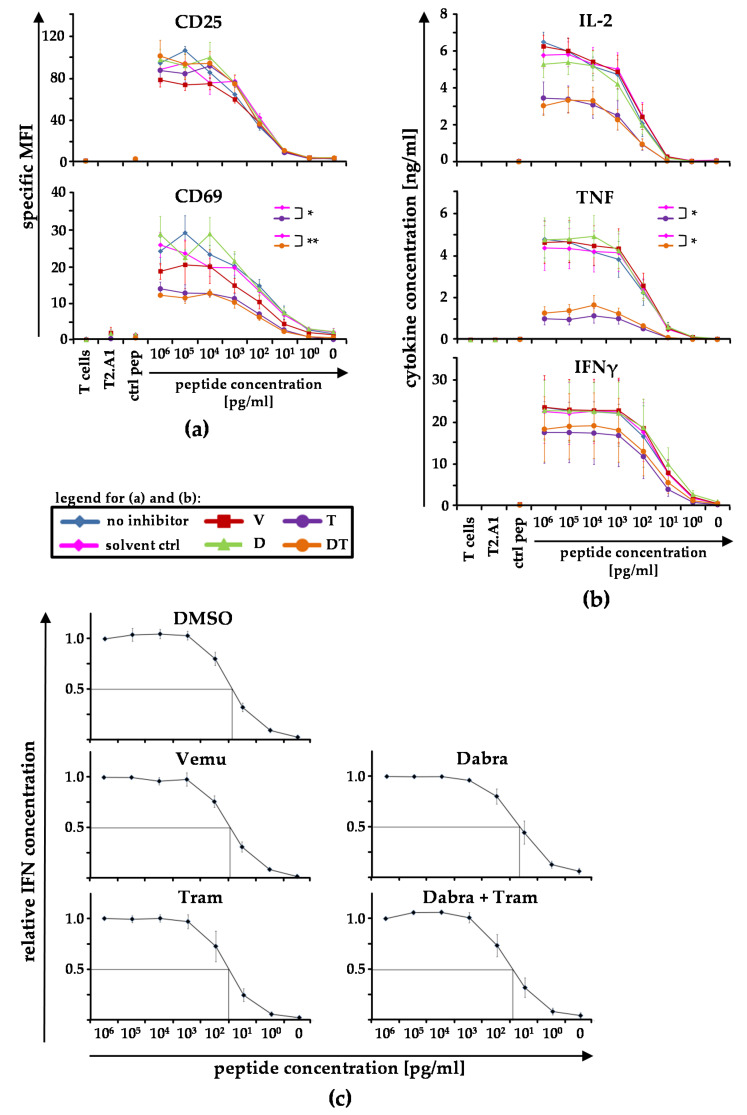
BRAF and MEK inhibitors do not affect T-cell avidity: CD8^+^ T cells were electroporated with RNA coding for a gp100-specific T-cell receptor (TCR). UV-irradiated T2.A1 cells were either left untreated (peptide concentration 0 pg/mL), loaded with control peptide MelanA analogue (ctrl pep), or loaded with the respective gp100 peptide at the indicated concentrations (10^6^−10^0^ pg/mL). T cells and T2.A1 cells were cultured at a 1:1 ratio in the presence of solvent control (DMSO), vemurafenib (V), dabrafenib (D), trametinib (T), or the clinically used combination D + T (DT) or without inhibitor treatment (no inhib). gp100-TCR-transfected CD8^+^ T cells (T cells) and irradiated T2.A1 cells (T2.A1) cultured alone or in the presence or absence of BRAF- or MEK-inhibitors, respectively, served as negative controls. After 17−20 h, cells (**a**) and supernatants were sampled (**b**,**c**). (**a**) Cells were stained for CD25 and CD69 expression and were subsequently analyzed by flow cytometry. The expression of both markers is depicted as specific MFI (i.e., MFI after subtraction of background MFI of the respective isotype control antibodies). (**b**) IL-2, TNF, and IFNγ concentrations in the supernatants were assessed by Cytometric Bead Array (CBA). (**a**,**b**) Mean values ± SEM of four donors are depicted. *p*-values were determined by two-way ANOVA. All the conditions were tested against the solvent control DMSO and are displayed in the respective graph. * *p* ≤ 0.05, ** *p* ≤ 0.01. (**c**) Relative IFNγ concentrations were calculated (normalized on the 10^6^ pg/mL data set) and are depicted for each inhibitor. Mean values ± SEM of six (DMSO), seven (vemu), and four donors (dabra, tram, and dabra + tram) are depicted. To quantify changes in the functional avidity, we determined the peptide concentrations, which corresponded with the half-maximal relative IFNγ concentrations (i.e., ED_50_; lines).

To check whether BRAFi and MEKi also influence T-cell stimulation, we performed initial experiments, in which we used some of the BRAFi/MEKi single or combination conditions, to investigate whether T-cell avidity is affected when the T cells were stimulated in the presence of the inhibitors (Figure 3). This was tested in assays detecting activation-marker expression and cytokine secretion profiles. Hence, we co-cultured UV-irradiated peptide-loaded T2.A1 cells with CD8^+^ T cells, which had been equipped with a gp100-specific TCR. We used varying concentrations of the gp100-peptide, ranging from 10^6^ pg/mL to 1 pg/mL, to pulse the T2.A1 cells. Non-peptide-loaded T2.A1 cells (0 pg/mL) and T2.A1 cells loaded with a control peptide (ctrl pep) served as negative controls. Additionally, T2.A1 cells and CD8^+^ T cells were incubated alone. Afterwards, the expression of CD25 and CD69 on T cells (Figure 3a), cytokine secretion by T cells (Figure 3b), as well as the ED_50_ of IFNγ secretion as indicator for the T-cell avidity (Figure 3c) were determined.

CD25 expression was not affected by BRAFi or MEKi, but the application of tram and D + T significantly compromised CD69 upregulation (Figure 3a). In contrast to the negative impact of vemu on DC maturation, vemu alone as well as dabra alone only slightly influenced the expression of CD69 on CD8^+^ T cells.

Assessing the effects of BRAFi/MEKi on cytokine secretion capabilities, tram and D + T significantly reduced TNF secretion (Figure 3b). The quantities of IL-2 and IFNγ were also reduced by tram and D + T, but the changes were not statistically significant. Again, vemu and dabra alone did not significantly change cytokine secretion.

To detect whether changes in the T-cell avidity occurred, we calculated the relative IFNγ concentration and determined the ED_50_ for each inhibitor (Figure 3c). No changes in the T-cell avidity were observed.

In conclusion, CD8^+^ T cells, which were stimulated by T2.A1 cells, were affected in their cytokine secretion capacity and their upregulation of CD69 expression by tram and D + T-treatment, but there was no influence on their functional avidity. These data encouraged us to investigate the influence of BRAFi/MEKi on antigen-specific stimulated T cells in more depth.

### 2.3. BRAFi and MEKi Prevent the Antigen-Specific Upregulation of Activation Markers on T Cells

To assess whether the observed changes in the expression pattern of CD69 (Figure 3a) can also be detected in CD4^+^ T cells, we performed the experiments as described before but used either CD8^+^ or CD4^+^ T cells for the co-cultivation with either non-peptide-pulsed or peptide-pulsed T2.A1 cells. Furthermore, we used the highest concentration of the gp100 peptide to pulse the T2.A1 cells.

**Figure 4:**
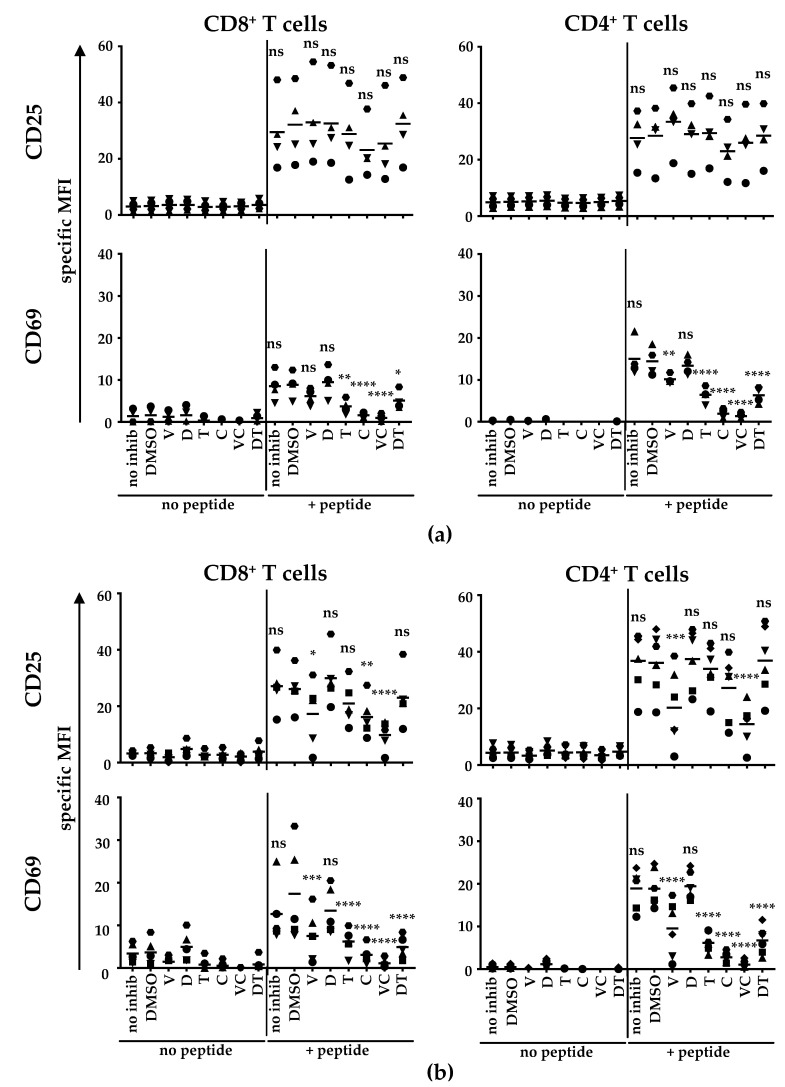
Cobi and V + C treatment almost completely inhibited the antigen-specific upregulation of the activation marker CD69 on both CD4^+^ and CD8^+^ T cells: CD8^+^ and CD4^+^ T cells were electroporated with RNA encoding a gp100-specific TCR. (**a**) UV-irradiated T2.A1 cells were left untreated or were loaded with the gp100 peptide. Afterwards, T2.A1 cells were co-incubated with CD8^+^ or CD4^+^ T cells at a 1:1 ratio in the presence of solvent control (DMSO), vemurafenib (V), dabrafenib (D), trametinib (T), cobimetinib (C), or the clinically used combinations V + C (VC) or D + T (DT) or without inhibitor treatment (no inhib). (**b**) moDCs were generated by plastic adherence, applying IL-4 and GM-CSF on days 1, 3, and 5. On day 6, cells were matured with IL-6, IL-1β, TNF, and PGE_2_. Cells were additionally treated without inhibitor (no inhib), with solvent control (DMSO), vemurafenib (V), dabrafenib (D), trametinib (T), cobimetinib (C), or the clinically used combinations V + C (VC) or D + T (DT). After 24 h hours, DCs were harvested and either left untreated (no peptide) or were loaded with the respective gp100 peptide (+peptide). Subsequently, CD8^+^ or CD4^+^ T cells were co-cultured with either unloaded or gp-100-loaded DCs at a 1:1 ratio in the presence of solvent control (DMSO), vemurafenib (V), dabrafenib (D), trametinib (T), cobimetinib (C), or the clinically used combinations V + C (VC) or D + T (DT) or without inhibitor treatment (no inhib). (**a**,**b**) After 17−20 h, cells were harvested, stained for CD25 and CD69 expression and were subsequently analyzed by flow cytometry. The expression of both markers on CD8^+^ and CD4^+^ T cells is depicted as specific MFI (i.e., MFI after subtraction of background MFI of the respective isotype control antibodies). Data of four (**a**) or six (**b**) donors (represented by different symbols) assessed in independent experiments are shown. Bars indicate mean values. *p*-values were determined by one-way ANOVA. In Dunnett’s multiple comparisons test, all conditions were tested against the solvent control DMSO. * *p* ≤ 0.05, ** *p* ≤ 0.01, *** *p* ≤ 0.001, **** *p* ≤ 0.0001, ns: *p* > 0.05.

As already shown in Figure 3a, BRAFi and MEKi did not influence CD25 expression on CD8^+^ T cells nor on CD4^+^ T cells (Figure 4a), but in contrast to CD8^+^ T cells, the expression of CD69 was significantly reduced by vemu on CD4^+^ T cells (Figure 4a). The antigen-specific upregulation of CD69 was inhibited both on CD8^+^ and CD4^+^ T cells by tram, cobi, D + T, and V + C treatment.

To determine the effects of BRAFi/MEKi during the interaction of CD4^+^ T cells and moDCs in an established in vitro licensing model [32], we performed co-cultures of moDCs with either CD8^+^ or CD4^+^ T cells (Figure 4b). For this purpose, moDCs were generated and co-incubated with BRAFi/MEKi either alone or in combinations during the maturation process. We intentionally used BRAFi/MEKi-pre-treated DCs for the stimulation of the T cells to mimic the natural situation in cancer patients under treatment conditions since both the DC maturation and the subsequent interaction of T cells would occur in the presence of the applied inhibitor. Thus, we co-cultured either gp100-TCR-specific CD8^+^ or CD4^+^ T cells with non-peptide-pulsed or peptide-loaded DCs that had been pre-treated with BRAFi/MEKi during maturation (accordingly to Figure 1 and Figure 2). It is of note that the gp100-specific TCR is of such high affinity that it is able to bind its complementary MHC-peptide complex when it is transferred to both CD8^+^ and CD4^+^ T cells.

Contrary to the effects seen on T cells after stimulation with T2.A1 cells (Figure 4a), vemu treatment clearly inhibited the upregulation of CD25 expression on both CD8^+^ and CD4^+^ T cells after antigen-specific stimulation with DCs (Figure 4b). Cobi alone also compromised CD25 expression on CD8^+^ T cells. This inhibition, induced by vemu, was more pronounced when vemu was combined with cobi treatment (Figure 4b). Neither dabra nor tram affected CD25 expression during the stimulation of CD8^+^ and CD4^+^ T cells with DCs (Figure 4b).

In contrast to the stimulation with T2.A1 cells, CD69 upregulation by CD8^+^ T cells after stimulation with DCs was clearly inhibited by vemu (Figure 4b). The inhibition induced by cobi, tram, V + C, and D + T was even stronger upon stimulation with DCs compared to stimulation with T2.A1 cells (Figure 4b). The CD4^+^ T cells behaved similarly after stimulation with DCs, i.e., CD69 expression was diminished after vemu, tram, cobi, V + C, and D + T-treatment. The vemu effect was more emphasized when they were stimulated with DCs than when they were stimulated with T2.A1 cells (Figure 4b). As already observed before, dabra did not impair CD25 and CD69 expression and did not change the inhibitory effects of tram.

In conclusion, the interaction of T cells and pre-treated moDCs represented a very sensible system, which was clearly affected by the administration of BRAFi and MEKi (with the exception of dabra treatment alone).

### 2.4. BRAFi and MEKi Suppress the Antigen-Specific Cytokine Secretion in T-Cell/DC Co-Cultures

**Figure 5:**
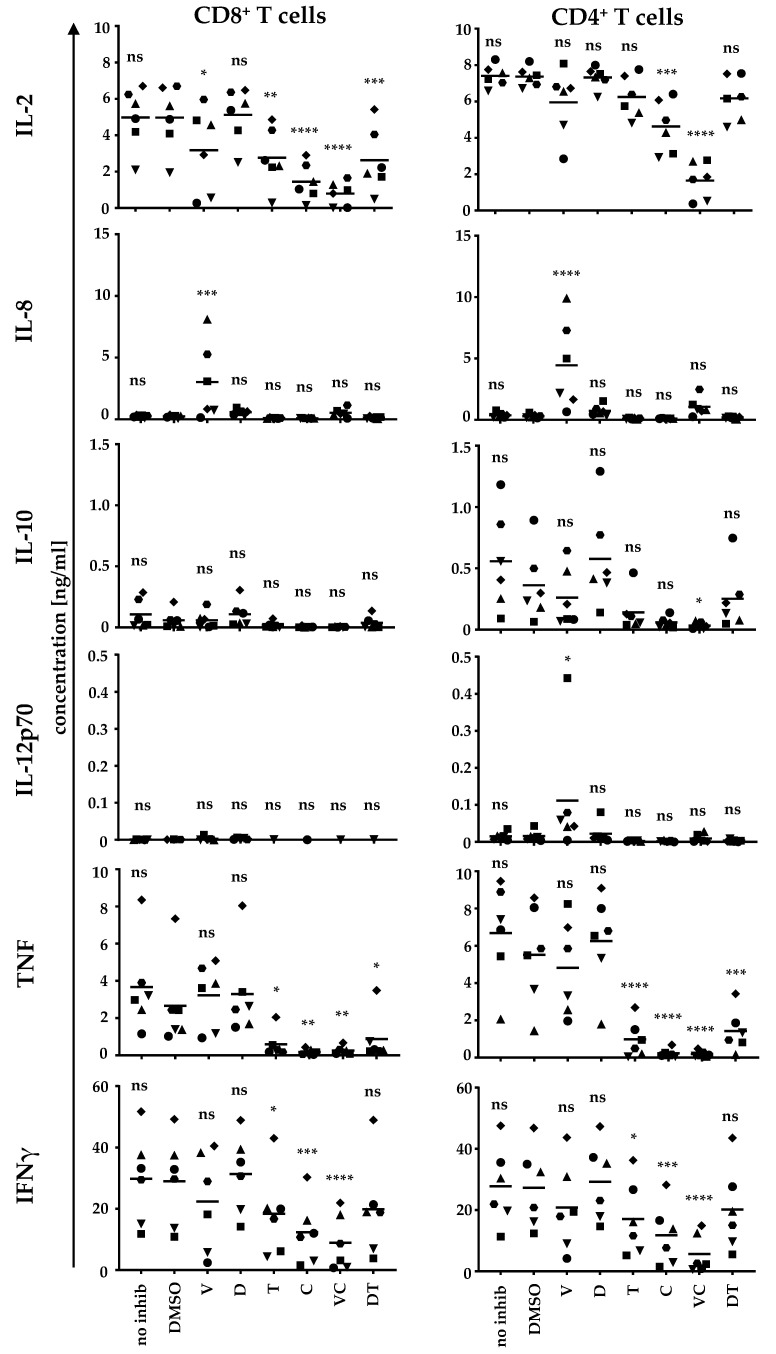
MEKi and specifically the combination of V + C severely compromise antigen-specific cytokine secretion by both CD8^+^ and CD4^+^ T cells upon stimulation: moDCs were generated and matured on day 6 with IL-6, IL-1β, TNF, and PGE_2_. During maturation, cells were additionally treated without inhibitor (no inhib), with solvent control (DMSO), vemurafenib (V), dabrafenib (D), trametinib (T), cobimetinib (C), or the clinically used combinations V + C (VC) or D + T (DT). After 24 h hours, DCs were harvested and were either left untreated (**see Figure S1**) or loaded with the respective gp100 peptide. gp100-TCR-transfected CD8^+^ and CD4^+^ T cells were subsequently co-cultured at a 1:1 ratio with either non-peptide-loaded or peptide-loaded DCs, again in the presence of solvent control (DMSO), vemurafenib (V), dabrafenib (D), trametinib (T), cobimetinib (C), the clinically used combinations V + C (VC) or D + T (DT), or without inhibitor treatment (no inhib), respectively. After 17–20 h, supernatants were collected, and cytokine secretion was assessed by CBA. Data from six donors (represented by different symbols) assessed in independent experiments are shown. Bars indicate mean values. *p*-values were determined by one-way ANOVA. In Dunnett’s multiple comparisons test all conditions were tested against the solvent control DMSO. * *p* ≤ 0.05, ** *p* ≤ 0.01, *** *p* ≤ 0.001, **** *p* ≤ 0.0001, ns: *p* > 0.05.

Since the expression of activation markers was severely inhibited upon BRAFi/MEKi treatment, we also investigated the impact on the cytokine secretion capability in T-cell/DC co-cultures (Figure 5 and Figure S1). We focused on the interaction with DCs as immunologically relevant cells. For this purpose, we co-incubated either CD8^+^ or CD4^+^ T cells equipped with a gp100-specific TCR and non-peptide-loaded (see Figure S1) or peptide-loaded moDCs (see Figure 5) in the presence or absence of BRAFi and MEKi and their combinations. Again, the DCs had been pre-treated with the BRAFi or MEKi either alone or in combination to mimic the situation in patients taking medication.

Vemu treatment induced a significantly increased IL-8 production (compared to the DMSO control co-culture) in co-cultures with both CD8^+^ and CD4^+^ T cells (Figure 5). This effect, however, was not caused by antigen-specific interaction, because co-cultures with non-peptide-loaded DCs showed very similar patterns (Figure S1). The secretion of all other cytokines required antigen-specific interaction because virtually no secretion was observed in the co-cultures with non-peptide-loaded DCs (Figure S1). Vemu also reduced IL- 2 release, but this primarily occurred for CD8^+^ T cells. For some donors, IL-2 secretion was completely abolished, whereas for other donors, IL-2 was barely affected (Figure 5). In contrast, IL-12p70 secretion was only increased in co-cultures with CD4^+^ T cells upon vemu treatment (Figure 5). Also here, a high variability was observed: vemu only raised IL-12p70 levels slightly for some donors, whereas large IL-12p70 quantities were detected for one donor. In contrast, dabra did not affect cytokine secretion capacities in the T-cell/DC co-cultures (Figure 5).

The MEKi tram and cobi significantly reduced the secretion of IL-2, TNF, and IFNγ in CD8^+^ and CD4^+^ T-cell/DC co-cultures (Figure 5), except for tram, which only affected IL-2 levels in co-cultures with CD8^+^ T cells. The cytokine reduction was more pronounced for the conditions in which cobi had been applied. The secretion of IL-2, TNF, and IFNγ was almost completely abolished when MEKi cobi was combined with BRAFi vemu (V + C). For TNF, reduction was significantly higher in co-cultures with CD4^+^ T cells (Figure 5). The reduction of TNF secretion was less severe in the D + T condition compared to the V + C condition, especially for CD8^+^ T cells (Figure 5).

In summary, BRAFi and MEKi clearly affected the cytokine secretion profile of T-cell/DC co-cultures, with the sole exception of dabra, which induced no significant changes. The combination of V + C almost completely abolished cytokine secretion.

### 2.5. V + C Treatment Affects Unspecific and Antigen-Specific CD8^+^ and CD4^+^ T-Cell Proliferation

The application of BRAFi and MEKi influenced both the expression of surface molecules on T cells and the cytokine secretion profile upon stimulation. Thus, we also investigated whether the proliferation of T cells was affected (unspecifically and after antigen-specfic stimulation by moDCs). Accordingly, we prepared co-cultures of either non-peptide-loaded (to detect the spontaneous proliferation) or peptide-loaded moDCs (to assess antigen-specific proliferation) and CD8^+^ (Figure S2, upper panel) or CD4^+^ T cells (Figure S2, lower panel). The T cells were labelled with CFSE dye to detect dilution upon cell division via flow cytometry. As described above, the moDCs were pre-treated with BRAFi and MEKi alone or in clinically used combinations. During co-cultivation, BRAFi and MEKi were applied as described above.

On day 3, co-cultures were harvested and examined by flow cytometry. The percentages of proliferated cells were analyzed in all conditions. We determined the spontaneous and the antigen-specific proliferation for each inhibitor treatment (Figure S2). The spontaneous proliferation of CD8^+^ T cells was only reduced by combined V + C treatment (Figure S2; upper panel), whereas the spontaneous proliferation of CD4^+^ T cells was diminished by vemu, tram, cobi, and V + C treatment (Figure S2; lower panel) (always compared to the DMSO control). Both, cobi and V + C application revealed a significantly decreased antigen-specific CD8^+^ T-cell proliferation (Figure S2; upper panel), while antigen-specific CD4^+^ T-cell proliferation was only attenuated by V + C supplementation (Figure S2; lower panel).

To sum up, even though vemu, tram, cobi, and V + C affected the spontaneous proliferation of CD4^+^ T cells and V + C that of CD8^+^ T cells upon interaction with moDCs, only the combination of V + C impaired the antigen-specific proliferation of CD4^+^ and CD8^+^ T cells.

### 2.6. BRAFi and MEKi Change the Surface Marker Expression Profile of MoDCs during Interaction with CD4^+^ and CD8^+^ T Cells

The antigen-specific interaction between DCs and CD4^+^ T cells is a bi-directional process and both cell types induce phenotypic changes in their respective counterpart [32]. Since we observed a change in the surface marker expression profile of T cells and in the cytokine secretion profile in co-cultures, we postulated that this would affect the changes in the moDCs phenotype, especially those induced with T-cell help. Therefore, we examined the surface marker expression of moDCs after antigen-specific interaction with the CD4^+^ T-helper in the presence of the inhibitors.

**Figure 6:**
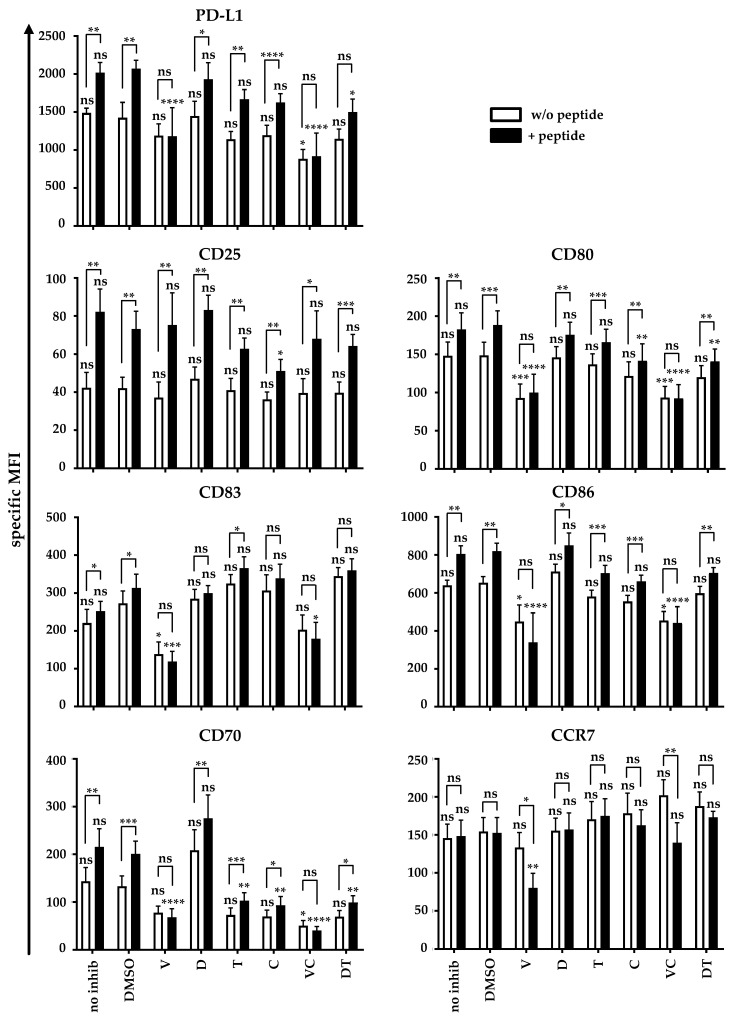
BRAFi and MEKi affect the upregulation of activation and maturation markers on moDCs upon stimulation with CD4^+^ T cells: CD4^+^ T cells were transfected with a gp100-specific TCR. moDCs were generated as described before and were substituted during the maturation process with solvent control (DMSO), vemurafenib (V), dabrafenib (D), trametinib (T), cobimetinib (C), the clinically used combinations V + C (VC) or D + T (DT), or without inhibitor (no inhib). After 24 h, DCs were either pulsed with the gp100 peptide (+ peptide, black bars) or were left untreated (w/o peptide, white bars). DCs and CD4^+^ T cells were co-cultured at a 1:1 ratio in the presence of solvent control (DMSO), vemurafenib (V), dabrafenib (D), trametinib (T), cobimetinib (C), the clinically used combinations V + C (VC) or D + T (DT), or without inhibitor treatment (no inhib). After 24 h, cells were harvested, stained for the indicated markers, and analyzed by flow cytometry. The expression of surface markers on the DCs is depicted as specific MFI (i.e., MFI after subtraction of background MFI of the respective isotype control antibodies). Data from six donors assessed in independent experiments are shown as mean values ± SEM. *p*-values were determined by one-way ANOVA or student’s t-test. In Dunnett’s multiple comparisons test, all conditions were tested against the solvent control DMSO for peptide and non-peptide conditions, respectively. To compare unspecific with antigen-specific surface marker expression, *p*-values were assessed by paired-student’s t-test for each inhibitor. * *p* ≤ 0.05, ** *p* ≤ 0.01, *** *p* ≤ 0.001, **** *p* ≤ 0.0001, ns: *p* > 0.05.

Thus, we again utilized our established licensing model [32] with the CD4^+^ T cells (Figure 6) as described above in the presence or absence of BRAFi and/or MEKi. Co-cultures without the addition of any substance and DMSO served as controls. Similar co-cultures were prepared with CD8^+^ T cells (Figure S3).

An influence of BRAFi and/or MEKi on the expression of distinct maturation and activation markers during the maturation process were detected on DCs (see Figure 2). These differences in phenotype carried through, thus influencing the values observed with non-peptide-loaded DC (Figure 6). Nevertheless, the antigen-specific interaction with the helper T cells further increased the expression levels of most maturation markers, and this process was differentially influenced by the different BRAFi/MEKi combinations.

PD-L1 expression increased significantly upon antigen-specific interaction. This was abolished by vemu, V + C, and to a lesser extent, by D + T, whereas V + C already reduced the expression in the condition without peptide (Figure 6). A significant antigen-specific increase in CD25 expression was observed in all conditions, which was significantly reduced, but not abolished by cobi treatment (Figure 6). The B7 proteins CD80 and CD86 behaved similarly. Their expression on moDCs was already reduced upon unspecific stimulation in the presence of vemu and V + C. The antigen-specific increase in CD80 and CD86 expression was completely inhibited by vemu and V + C. Additionally, CD80 expression was partially inhibited by cobi and D + T (Figure 6). CD83 displayed a slight but significant antigen-specific increase in expression in the absence of the inhibitors. Vemu and V + C resulted in decreased CD83 expression on the moDCs in stimulations without peptide and also completely abolished the antigen-specific increase upon stimulation (Figure 6). CD70 expression had already appeared to be very sensitive for MEKi and BRAFi treatment during maturation (Figure 2). Hence, we observed the reduced expression of CD70 on moDCs in unspecific conditions under the influence of all inhibitors except of dabra (Figure 6). Treatment with the inhibitors also compromised the antigen-specific upregulation of CD70, either completely (vemu, V + C) or partially (tram, cobi, D + T) (Figure 6). Treatment with dabra alone did not affect the antigen-specific increase in CD70 expression (Figure 6). CCR7 was not induced antigen-specifically in the absence of inhibitors, but in the presence of vemu and V + C, its expression dropped significantly upon antigen-specific stimulation (Figure 6).

Thus, BRAFi and MEKi clearly affect the upregulation of surface markers and hence also the activation of moDCs by T-helper cells and thereby the immune response. The addition of antigen-specific T-helper cells could not overcome the negative impact of the inhibitors and their combinations. As described above, the addition of D + T resulted in much weaker effects than V + C.

To test whether the interaction of DCs and CD8^+^ T cells was also affected by BRAFi and MEKi, we also performed co-cultures of pre-treated moDCs and CD8^+^ T cells (Figure S3). Since moDCs were not activated by CD8^+^ T cells [32], the analysis yielded results that were very similar to those of CD4^+^ T cells (Figure 6); however, the activating effects of the CD8^+^ T cells on the DCs were weaker (Figure S3).

In conclusion, BRAFi and MEKi do affect DC maturation, T-cell stimulation, and T-cell proliferation, and also change the expression profile on moDCs in T-cell co-cultures. Furthermore, they clearly change cytokine secretion patterns. Thus, BRAFi and MEKi clearly and severely affect the immune cells and their function and hence the immune response. Even though dabra and tram also had a negative impact on the described immunological processes, their effects were much less severe than those observed with the administration of vemu and cobi, and especially the combination of V + C.

Thinking of a possible combination of cellular therapy and BRAFi/MEKi treatment, our results suggest the use of the combination of D + T since this combination showed less inhibitory effects on the DCs and T cells.

## 3. Discussion

A better understanding of the effects of kinase inhibitors on normal immune cell function is required for a reasonable concurrent application of a combination of BRAFi/MEKi with immunotherapy in the treatment of cancer patients. In this study, we investigated the influence of the commonly used BRAFi/MEKi on moDCs by determining the effects on cytokine secretion by DCs and the phenotype of DCs, when the inhibitors were added directly during the maturation process. Furthermore, we examined the influence of BRAFi/MEKi on DC/T-cell interactions by determining the cytokine secretion pattern and the T-cell and DC phenotype after antigen-specific bi-directional interaction. We clearly observed the negative effects of these inhibitors, which were the most pronounced for vemu, cobi, and the combination of both, and were much less observed for dabra, tram, and the combination D + T.

Both vemu and dabra are selective type 1 BRAF, adenosine triphosphate-competitive inhibitors, which are chemically similar but not identical (i.e., vemu: C_23_H_18_ClF_2_N_3_O_3_S, dabra: C_23_H_20_F_3_N_5_O_2_S_2_; chemical structure is depicted in Heinzerling et al. [33]). As described, both have a proven efficacy in BRAF^V600E^ metastatic melanoma and have a similar clinical activity and class-defined toxicity. However, there are some differences in RAF kinase inhibition. The drug concentration required for 50% inhibition of the kinase activity (IC_50_) of vemu for BRAF^V600E^ is 31 nM. Additionally, vemu has a similar IC_50_ (i.e., 48 nM) for CRAF inhibition. This is not the case for dabra (IC_50_: 0.6 nM and 5 nM, respectively) [11,34]. Furthermore, dabra is a more selective inhibitor for BRAF^V600E^ than vemu, as indicated by the ratio of IC_50_ for BRAF^V600E^ vs. BRAF^wt^, which is 0.3 for vemu [11] and 0.05 for dabra [34]. Moreover, dabra has a similar potency for the inhibition of BRAF^V600E^ and BRAF^V600K^ [35].

Likewise, both cobi and tram are reversible inhibitors of MEK1 and MEK2, blocking both their activation and kinase activity, which are chemically similar but not identical (i.e., cobi: C_21_H_21_F_3_IN_3_O_2_, tram: C_26_H_23_FIN_5_O_4_ [33]). The chemical differences between the BRAFi/MEKi probably are also the cause for the different elimination half-lives (i.e., 56 h vs. 8.4 h for vemu and dabra, respectively, and 44 h vs. 90 h for cobi and tram, respectively [33]).

Especially the differences in RAF kinase inhibition between vemu and dabra and the stronger impact of the former on the wild-type BRAF, which may result in a stronger impact on the immune cells, can explain the differential observations we made in our in vitro study.

### 3.1. BRAFi and MEKi Effects on T Cells

We have assessed the effects of BRAFi and MEKi in single treatment or in combination on T-cell stimulation in our already validated in vitro model system, which consists of moDCs and TCR-transfected T cells to observe antigen-specific interaction [32]. Other groups have analyzed the effects on T-cell function for some of the inhibitors, e.g., Boni et al. utilized BRAFi (PLX4720) and MEKi (U0126 and PD0325901) [30]. In line with our data, the MEKi impaired T-cell function in terms of IFNγ production, but in contrast to our data, they observed reduced T-cell viability [30]. Similar to what we observed for dabra, the specific BRAF^V600^ inhibitor PLX4720 did not affect T-cell function [30], although it is chemically more closely related to vemu. However, they used IL-2/OKT-3-expanded lymphocytes from patients and analyzed proliferation as well as the recognition of tumor cell lines by virally TCR-transduced T cells.

Others detected an inhibition of stimulated CAR-transduced T cells only when vemu, dabra, or tram were applied at high concentrations in vitro [28]. CD25 expression was reduced upon treatment with high inhibitor concentrations when the cells were stimulated by CD3/CD28 but was also diminished due to intermediate concentrations of vemu, tram, and D + T [28]. The combination of D + T inhibited T-cell proliferation and effector functions at low inhibitor concentrations. Dabra alone had little adverse effects on CAR T-cell function [28]. The authors, therefore, suggest using dabra alone as an alternative for combinatory CAR-T-cell therapy [28]. We also detected effects on CD4^+^ and CD8^+^ T-cell proliferation and were able to assess a negative impact of vemu, tram, cobi, and V + C but not of D + T on the spontaneous proliferation of CD4^+^ T cells. CD4^+^ T-cell proliferation upon antigen-specific stimulation was exclusively inhibited by V + C. Spontaneous CD8^+^ T-cell proliferation was only affected by V + C treatment, while antigen-specific proliferation was also affected by cobi. In contrast to Gargett et al., we did not detect a negative impact of D + T on T-cell proliferation. Nevertheless, in line with their data, we also detected a reduction in CD25 expression. However, this was limited to vemu, cobi, and V + C for CD8^+^ T cells and vemu and V + C for CD4^+^ T cells. We did not see an effects by tram or D + T. We only detected changes in CD69 expression after the treatment of the CD4^+^ and CD8^+^ T-cell stimulations with vemu, tram, cobi, V + C, and D + T. These differences could have been caused by the fact that we used moDCs to stimulate the TCR-transfected T cells, whereas Gargett et al. used CAR-T-cells or CTLs expanded from PBMCs.

Liu et al. reported a reduced CD4^+^ T-cell proliferation upon CD3/CD28 stimulation by the exposure to tram and D + T at concentrations above 0.1 µM [36]. We did not detect such effects, probably because we used lower concentrations of tram, which were corresponding to the concentrations found in patient plasma. Liu et al. observed a partial inhibition of IL-2, TNF, and IL-8 by tram and an induction of IL-4 secretion [36]. After D + T treatment, the tram-induced effects seemed to dominate and were minimized when the CD4^+^ T cells were first activated [36]. We also detected an inhibitory effect of tram and D + T on IL-2 secretion in CD8^+^ T-cell co-cultures, on TNF secretion in CD8^+^ and CD4^+^ co-cultures, and of tram on IFNγ production in CD8^+^ and CD4^+^ co-cultures. In contrast to us, Liu et al. did not find an effect of tram or D + T on CD69 expression but detected the inhibition of CD25 expression by tram before activation, findings that were also subsequently determined by D + T [36].

Vella et al. investigated the effects on T lymphocyte function and on moDC surface marker expression [26]. Dabra had no impact on T lymphocytes or moDCs. Tram alone or in combination with dabra suppressed T-lymphocyte proliferation, cytokine production, and antigen-specific expansion [26], which corroborates some of our data. We also detected a negative influence of D + T on TNF and IFNγ secretion in T-cell/moDC stimulations and a lower CD69 expression on T cells after moDC stimulation, even though the negative impact was more intensified upon V + C treatment, which was also included in our study.

### 3.2. BRAFi and MEKi Effects on DCs

Regarding the effects on moDCs, Vella et al. detected an induction of DC maturation, which could be measured by CD83 and CD86 expression, when using LPS-matured moDCs generated from CD14^+^ monocytes upon treatment with tram and D + T [26]. Probably because we matured our moDCs in the presence of the common cytokine maturation cocktail, consisting of IL-6, TNF, PGE_2_, and IL-1β, we did not observe an induction of DC maturation but an inhibition of distinct maturation markers upon D + T treatment (i.e., CD80, CD86, CD70) and an induction of CCR7 expression. Again, the observed inhibitory effects were more enhanced by V + C treatment.

Other groups have also tested the effect of BRAFi and MEKi on DCs (reviewed in [13]). Hajek et al. showed that murine bone marrow-derived (BM)-DCs increased the expression of CD80 and CD86 after treatment with dabra, D + T, and V + C, and of MHC cl. II after treatment with dabra, tram, cobi, and D + T [37]. In addition, IL-1β secretion by LPS-stimulated BM-DCs was induced upon treatment with dabra, D + T, and vemu. The combination of V + C completely abrogated IL-1β secretion. Production of other cytokines (e.g., TNF, IL-12) was reduced after treatment with dabra and D + T but was not influenced by cobi or V + C [37]. Treatment with vemu alone elevated IL-12 secretion. Furthermore, the viability of BM-DCs was clearly reduced after treatment with dabra, D + T, vemu, cobi, and V + C [37]. The same group showed that dabra and vemu upregulated the CD80 expression on human day 6, unstimulated, moDCs [37]. In contrast to the IL-1β secretion by BM-DCs observed by Hajek et al., we never found an increase in IL-1β secretion by our human monocyte-derived, cocktail-matured DCs (data not shown). This may be due to technical reasons since IL-1β is part of our maturation cocktail, and additional IL-1β secreted by the moDCs is difficult to detect. In line with Hajek et al., we also observed an increase in IL-12 secretion with the addition of vemu alone and no difference when V + C or tram alone were added. However, we did not observe a reduction in IL-12 production by dabra or D + T, even when using similar inhibitor concentrations. We also never saw differences in TNF secretion (data not shown), but similar to IL-1β, TNF was part of the maturation cocktail, so changes in TNF levels might not have been detected. Considering CD80 and CD86 expression, we saw a reduced upregulation during maturation with vemu, dabra (only CD80), tram, cobi, V + C, and D + T. The difference in the data with BM-DCs and human moDCs of Hajek et al. may have been caused by the absence of LPS stimulation and maturation, respectively. We did not determine MHC cl. II expression. Partially in line with this, we also saw a reduced viability after treatment with vemu and V + C.

Ott et al. investigated the effects of vemu and a MEKi called U0126 on human monocyte-derived, polyI:C-matured DCs [38]. They observed the reduced production of IL-12 and TNF after treatment with a wide range of U0126 concentrations and after treatment with their highest concentration (i.e., 50 µM) of vemu [38]. Furthermore, they observed the downregulation of CD83 and CD80 after treatment with the MEKi but not with (low concentration; 1 µM) vemu [38]. The cytokine secretion data of Ott et al. is contradictory to our cytokine data (i.e., upregulation of IL-12 production and no change in TNF production after treatment with vemu) and might be explained by the different maturation stimuli that were used (polyI:C versus cytokine cocktail) and the slightly different inhibitor application timing (i.e., 24 h pre-treatment with inhibitor and subsequent 24 h maturation by Ott et al., versus 24 h simultaneous inhibitor treatment and maturation in our study to mimick the physiological situation). We did not observe a reduction in the upregulation of CD83 after treatment with tram and cobi, which was observed with U0126. However, since these are different molecules, they are hard to compare. For CD80 we also saw a lower expression after treatment with MEKi. The absence of the negative effect of vemu treatment on CD80 and CD83 expression in the study of Ott et al. may be caused by the very low concentration that they used (i.e., 1 µM versus 60 µM to simulate the physiological plasma levels in our study).

Tel et al. showed that ex vivo-isolated plasmacytoid DCs (pDCs) and myeloid DCs (mDCs) of healthy donors, which were matured with R848, had a reduced expression of CD80 (pDCs and mDCs) and CD86 (pDCs) after treatment with vemu [39]. This is perfectly in line with our data. Furthermore, they measured a lower TNF production by pDCs after vemu treatment [39], which was not the case in our study (which is probably due to the inability to measure small changes in TNF concentration, as explained above). Dabra, tram, and D + T did not influence the expression of CD80 and CD86 on pDCs and mDCs [39], for which we saw a downregulation in our study with moDCs. Interestingly, Tel et al. also observed a negative effect of vemu on CD69 expression on T cells after antigen-specific stimulation with peptide-loaded pDCs and mDCs [39].

Finally, Riegel et al. studied the effects of a panRAF inhibitor (i.e., inhibiting BRAF^V600^ mut, BRAF^wt^, and CRAF^wt^) and tram on human LPS-matured moDCs [40]. Because the mode of action of the panRAF inhibitor is largely different from that of vemu and dabra, these cannot be compared. Nevertheless, these authors detected an upregulation of CD83 and no effect on CD80 expression after treatment with tram [40]. Notably, they observed an upregulation of CCR7 after treatment with tram, as did we [40]. Considering cytokine production, they measured no differences in IL-12p70 secretion and lower secretion of TNF and IL-8 after treatment with tram [40]. We did not observe any differences in the secretion of these three cytokines. Both the differences in surface marker expression and cytokine secretion might be caused by the different maturation stimuli used or the different concentration of tram utilized (1 µM in Riegel et al. versus the concentration found in plasma of 30 nM). Proliferation of CD4^+^ T cells induced by antigen-unspecific stimulation with anti-CD3 and anti-CD28 mAbs was clearly inhibited by tram [40]. This is in contrast with the proliferation assay we performed in an antigen-specific setting, in which we did not see an effect of tram.

Even though we detected more pronounced negative effects using BRAFi/MEKi combinations than single substances, the combinations constitute the standard treatment for patients with BRAF^V600^ mutant melanoma because of superior objective response rates and progression-free survival rates in D + T-treated patients compared to dabra-only treated patients observed in clinical trials [10,15,41]. As reviewed in [10], the D + T and V + C treatment combinations were almost equal concerning clinical effectivity data. Since we detected weaker negative effects on both DC maturation and T-cell activation induced by D + T, we suggest prioritizing D + T when a combination with other immunotherapies is considered.

### 3.3. BRAFi and MEKi: Implications for Combination Therapies

In our opinion, the findings presented in this study are of eminent importance for potential combination therapies with BRAFi/MEKi and checkpoint inhibitor therapy. Any combination attempt using cell-based therapies plus targeted therapy, including DC vaccination in the treatment of melanoma, needs to carefully address the potential immunosuppressive effects of these drugs. However, these findings can have an even wider impact. With respect to clinical trials for melanoma patients, as listed in clinicaltrials.gov (accessed on 7 October 2021), many studies combine BRAFi/MEKi with checkpoint inhibitors (CPI), such as pembrolizumab, nivolumab (both targeting PD-1), ipilimumab (targeting CTLA-4), or avelumab and atezolizumab (both targeting PD-L1) (NCT02818023, NCT03625141, NCT04722575, NCT02908672 NCT03554083, NCT02902029, NCT03149029, NCT02910700, NCT02858921, NCT01940809). The application of these CPI results in “releasing the brakes” on T cells for their effective priming against certain tumor antigens [42]. In this priming process, DCs play a pivotal role. The co-application of BRAFi/MEKi that has a deleterious effect on DCs might negatively affect the priming process. Furthermore, the effects of BRAFi/MEKi on the reciprocal interaction of DCs and T-helper cells (shown in this study) might subsequently abolish DC activation and impede optimal CTL stimulation since mutual interplay was shown to be efficient for full CTL induction [32]. It was already published by Liu et al. that tram in concurrent application with anti-PD-1 downregulated immunosuppressive factors or upregulated HLA molecules. The combination of tram and anti-PD-1 led to an increased infiltration of lymphocytes and resulted in a decreased tumor volume and increased survival of the mice [36]. All three combinatory settings (PD-1 1st/MEKi 2nd, MEKi 1st/PD-1 1st, MEKi 1st/PD-1 2nd) showed tumor growth inhibition that was more effective than single-agent treatments, but concerning the survival of the mice, the last two combinations were much better (MEKi 1st/PD-1 1st, MEKi 1st/PD-1 2nd). Hence, MEKi should be given first, whereas anti-PD1 might be applied concomitantly or later on [36]. Additionally, the combination of tram and anti-PD1 led to increased lymphocyte infiltration [36].

Another example is the combined use of BRAFi/MEKi and oncolytic viruses such as Talimogene laherparepvec (T-Vec; NCT03088176). These oncolytic viruses are injected directly into the tumor, causing the local destruction of virus-infected tumor cells, subsequently resulting in a systemic immune response against other metastases induced by DCs and other immune cells [43]. The choice of BRAFi/MEKi in combination with T-Vec may be essential here.

The SARS-CoV-2 pandemic impressively showed the effectiveness of mRNA-vaccine strategies. In fact, mRNA-vaccine development was initiated to treat cancer. After the success with different mRNA-based COVID-19 vaccines, many companies now have mRNA-based therapeutic cancer vaccines in their development pipelines once again, which probably will enter late phase clinical trials soon. With respect to these novel approved therapies, the observations presented in this study are of importance. Combination therapies of BRAFi/MEKi with mRNA-vaccines are an obvious way to go. However, the effectiveness of mRNA-based vaccines largely depends again on DCs, and therefore, the effectiveness of a combination therapy may be subject to the correct choice of BRAFi/MEKi.

In summary, the choice of BRAFi/MEKi agents should be carefully made when combined with other immunotherapies since it can have a detrimental consequence on the effectiveness of the anti-cancer immune response.

## 4. Materials and Methods

### 4.1. BRAF and MEK Inhibitors

Vemurafenib was acquired from Adooq Bioscience (Irvine, CA, USA), dabrafenib from AbMole BioScience (Housten, TX, USA), trametinib from Selleckchem (Housten, TX, USA), and cobimetinib from Roche (Basel, Switzerland). BRAF and MEK inhibitors were diluted according to the manufacturers’ instructions with DMSO (Life Technologies, Darmstadt, Germany). Final concentrations of the inhibitors are shown in Table 1.

### 4.2. Cells and Reagents

Monocyte-derived DCs were generated from the fresh blood of healthy volunteers following informed consent and approval by the institutional review board (Ethikkommission of the Friedrich-Alexander University Erlangen-Nürnberg, Ref. no. 43_15 B) as described previously [44]. PBMCs were purified by density centrifugation (Lymphoprep, Axis-Shield PoC AS, Oslo, Norway). Monocytes were separated from the non-adherent fraction (NAF) by plastic adherence. Monocytes were applied with 275 U/mL IL-4 (Miltenyi Biotec, Bergisch Gladbach, Germany), 800 U/mL GM-CSF (Miltenyi Biotec, Bergisch Gladbach, Germany), and DC medium (consisting of RPMI 1640 (Lonza, Verviers, Belgium) containing 1% heat-inactivated human AB serum (Sigma-Aldrich, St. Louis, MO, USA), 2 mM L-glutamine (Lonza, Verviers, Belgium), and 20 mg/L gentamicin (Lonza, Verviers, Belgium)) on days one, three, and five. DCs were matured for 24 h on day 6 with 200 IU/mL IL-1β (CellGenix, Freiburg, Germany), 1000 U/mL IL-6 (Miltenyi Biotec, Bergisch Gladbach, Germany), 10 ng/mL TNF (Beromun, Boehringer Ingelheim Pharma, Ingelheim am Rhein, Germany), and 1 μg/mL PGE_2_ (Pfizer, Zurich, Switzerland).

T cells (CD4^+^ and CD8^+^) were isolated from the non-adherent fraction (NAF) using MACS beads (Miltenyi Biotec, Bergisch Gladbach, Germany), according to the manufacturer’s instructions. Subsequently, T cells were cultured in MLPC medium consisting of RPMI 1640 (Lonza, Verviers, Belgium), 10% human AB serum (Sigma-Aldrich, St. Louis, MO, USA), 2 mM L-glutamine (Lonza, Verviers, Belgium), 20 mg/L gentamycin (Lonza, Verviers, Belgium), 10 mM HEPES (PAA Laboratories, GE Healthcare Life Sciences, Pasching/Linz, Austria), 1 mM sodium pyruvate (Lonza, Verviers, Belgium), and 1% MEM nonessential aa (100×, Lonza, Verviers, Belgium), supplemented with 10 ng/mL IL-7 (Peprotech, Hamburg, Germany) and 5 ng/mL IL-15 (R & D Systems, Minneapolis, MN, USA) (CD4^+^ T cells) or 10 ng/mL IL-7 (CD8^+^ T cells).

T2.A1 cells (TAP-deficient TxB cell hybrid) were cultured in R10 medium consisting of RPMI 1640 (Lonza, Verviers, Belgium) supplemented with 2 mM L-glutamine (Lonza, Verviers, Belgium), penicillin–streptomycin (Lonza, Verviers, Belgium), 10% fetal calf serum (PAA Laboratories, GE Healthcare Life Sciences, Pasching/Linz, Austria), 2 mM HEPES (PAA Laboratories, GE Healthcare Life Sciences, Pasching/Linz, Austria), and 2 mM 2-mercaptoethanol (Gibco, Life Technology, Carlsbad, CA, USA).

### 4.3. MoDC Pre-Treatment

moDCs were generated and matured as described above. Concomitantly with the maturation cocktail, cells were either treated with vemurafenib (vemu), dabrafenib (dabra), trametinib (tram), cobimetinib (cobi), the combination of vemu and cobi (V + C), the combination of dabra and tram (D + T), DMSO solvent control, or were left untreated. Inhibitor concentrations are described in Table 1. After 24 h, cells were harvested and either directly analyzed or utilized in further experimental setups.

### 4.4. In Vitro Transcription and Electroporation of CD4^+^ and CD8^+^ T Cells

RNA encoding the gp100/HLA-A2-specific TCR was generated using the in vitro transcription kit with T7 RNA polymerase (mMESSAGE mMACHINE kit; Ambion, ThermoFisher Scientific, Darmstadt, Germany), according to the manufacturer’s instructions as described before [44]. RNA coding for the gp100-specific TCR was electroporated into CD4^+^ or CD8^+^ T cells as previously described [45,46].

### 4.5. Co-Cultivation of CD4^+^ or CD8^+^ T Cells with T2.A1 Cells or MoDCs

moDCs were generated, matured, and pre-treated as described above. T2.A1 cells were UV-irradiated (0.005 J/cm^2^). moDCs and T2.A1 cells were loaded for 1 h at 37 °C with the indicated peptides (10 μg/mL) or were left untreated. CD4^+^ and CD8^+^ T cells were electroporated with a gp100-specific TCR as described above. Four hours after electroporation, T cells were either co-cultured with moDCs or T2.A1 cells at a 1:1 ratio. Concomitantly, BRAFi and/or MEKi were applied at the indicated concentration (Table 1). DMSO served as solvent control. A second control condition was conducted completely without the application of any further substance.

### 4.6. Peptide Titration Assay

UV-irradiated and peptide-loaded T2.A1 cells were co-cultured with CD8^+^ T cells, which had been equipped with a gp100-specific TCR. To determine the changes in the avidity, varying concentrations of the gp100-peptide, ranging from 10^6^ pg/mL to 10^0^ pg/mL, were utilized to pulse the T2.A1 cells. BRAFi and/or MEKi were applied at the indicated concentration (Table 1).

### 4.7. CFSE Proliferation Assay

CD4^+^ and CD8^+^ T cells were electroporated with RNA coding for the gp100-specific TCR and were subsequently labelled with CFSE according to the Cell Trace proliferation kit (Invitrogen, ThermoFisher Scientific, Darmstadt, Germany) using anhydrous DMSO. Co-cultures between T cells and peptide-loaded or non-peptide-loaded moDCs was performed as described above at a 1:1 ratio. BRAF and/or MEK inhibitors were applied at the indicated concentration (Table 1). After three days, the cells were harvested and were analyzed using flow cytometry. To assess antigen-specific vs. the spontaneous proliferation, we calculated the percentage of proliferated CD8^+^ or CD4^+^ T cells either after antigen-specific (gp100 peptide) or unspecific (no peptide) stimulation, respectively.

### 4.8. Cytokine Analysis

Cytokine concentrations in the co-culture supernatants were analyzed after 17−20 h with the Inflammatory Cytometric Bead Array (BD Biosciences, Heidelberg, Germany) and a Th1/Th2 Cytometric Bead Array (BD Biosciences, Heidelberg, Germany) following the manufacturer’s instructions.

### 4.9. Surface Marker Expression Analysis

Extracellular surface marker staining was performed with IgG1-FITC, IgG2a-FITC, IgG1-PE, αCD25-FITC, αCD40-PE, αCD69-PE, αCD70-PE, αCD80-FITC, αCD83-PE, αCD86-FITC, and PD-L1-PE (all from BD Biosciences, Heidelberg, Germany); IgG3-PE (eBioscience, Frankfurt, Germany); and αCCR7-FITC (R&D Systems, Minneapolis, MN, USA) for 30 min at 4 °C in PBS supplemented with 1% FCS and 0.02% sodium azide (Merck, Darmstadt, Germany). The cells were analyzed using a FACScan cytofluorometer equipped with CellQuest software (BD, Heidelberg, Germany). Analysis was performed with the FCS Express software (De Novo Software, Glendale, CA, USA). Specific MFIs were calculated by subtracting the background MFI obtained with the isotype controls.

### 4.10. Statistical Analysis

Statistical analysis was performed using Graph Pad Prism software (LLC: San Diego, CA, USA). *p*-values were determined by 1-way ANOVA without greenhouse correction. In Dunnett’s multiple comparisons test, all conditions were tested against the solvent control DMSO. To compare unspecific with antigen-specific surface marker expression (Figure 6 and Figure S3) or the proliferation of the T cells (Figure S2), *p*-values were assessed by a paired-student’s t-test for each inhibitor. * *p* ≤ 0.05, ** *p* ≤ 0.01, *** *p* ≤ 0.001, **** *p* ≤ 0.0001, ns: *p* > 0.05.

To compare surface expression or cytokine secretion kinetics between non-peptide-loaded and peptide-loaded conditions (Figure 3), a two-factor analysis of variance (two-way ANOVA) was used to determine the interaction *p*-value between the whole curves * *p* ≤ 0.05, ** *p* ≤ 0.01, *** *p* ≤ 0.001, **** *p* ≤ 0.0001, ns: *p* > 0.05.

## 5. Conclusions

Taken together, we present here a broad analysis of effects of BRAFi/MEKi on monocyte-derived DCs, their stimulatory capacity, and their bi-directional interaction with T-helper cells using clinically relevant concentrations (physiological concentrations found in plasma after treatment) and combinations of these inhibitors. This study shows that BRAFi/MEKi influence immune function. Since these influences are highly dependent on the type of inhibitor, one must carefully consider the differential effects in the choice of combination trials. Considering the data presented above, we suggest that DC vaccination therapy, and other therapies involving DCs, for that matter, should be combined with D + T rather than with V + C.

## Figures and Tables

**Table 1:** Final concentrations and respective targets of BRAFi/MEKi utilized in the experiments.

Inhibitor/ Inhibitor Combinations	Final Concentration	Target
vemurafenib (vemu, V)	60 µM	BRAF^V600E^
dabrafenib (dabra, D)	1 µM	BRAF^V600E^
trametinib (tram, T)	30 nM	MEK 1/2
cobimetinib (cobi, C)	0.5 µM	MEK 1/2

## Data Availability

All original data are available from the corresponding author upon request.

## Supplementary Materials

The following are available online at https://www.mdpi.com/article/10.3390/ijms222111951/s1.
