# Supplementary material for: BRAF and MEK Inhibitors Affect Dendritic-Cell Maturation and T-Cell Stimulation
Source: Int J Mol Sci. 2021 Nov 4;22(21):11951. doi: 10.3390/ijms222111951 (PMC8585071; doi:10.3390/ijms222111951)
Supplement: Supplementary file 1 [file ijms-22-11951-s001.zip › ijms-1432596-supplementary.pdf]

Supplemental figures

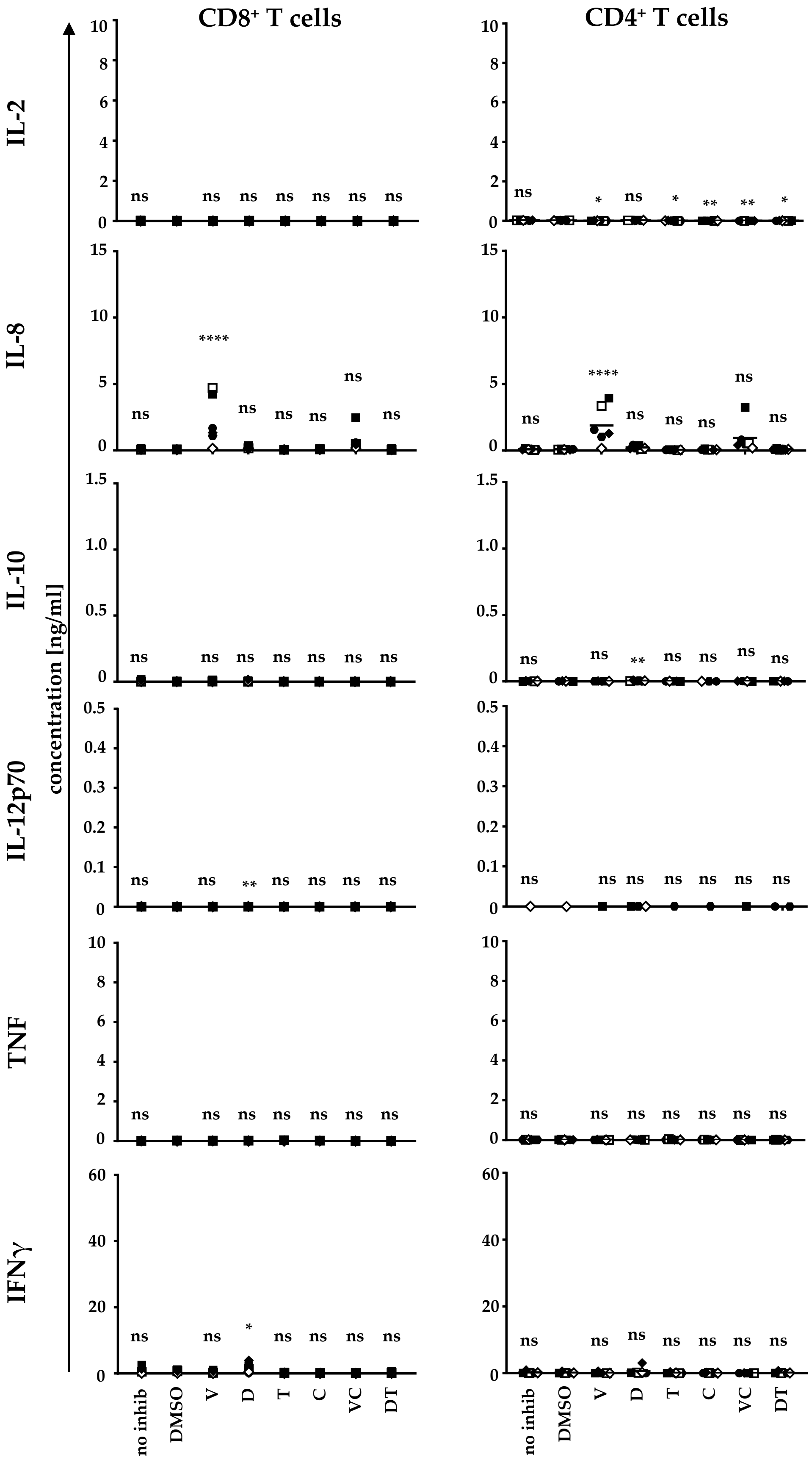

Figure S1. $CD8^+$ and $CD4^+$ T cells do not secrete cytokines after unspecific stimulation with DCs, except for IL-8 upon vemurafenib-treatment: moDCs were generated and were matured on day 6 with IL-6, IL-1β, TNF, and PGE_2.

Cells were additionally treated without inhibitor (no inhib), with solvent control (DMSO), vemurafenib (V), dabrafenib (D), trametinib (T), cobimetinib (C), or the clinically used combinations VC or DT. After 24h hours, DCs were harvested and either left untreated (depicted in this figue) or were loaded with the respective gp100 peptide (**see Figure 5**). gp100-TCR-transfected $CD8^+$ and $CD4^+$ T cells were subsequently co-cultured at a 1:1 ratio with these either non-peptide-loaded or peptide-loaded DCs, in the presence of solvent control (DMSO), vemurafenib (V), dabrafenib (D), trametinib (T), cobimetinib (C), or the clinically used combinations VC or DT, or without inhibitor treatment (no inhib). After 17-20 hours, supernatants were taken and cytokine secretion was assessed by CBA. Data of six donors (represented by different symbols) assessed in independent experiments are shown. Bars indicate mean values. p-values were determined by one-way ANOVA. In the Dunnett's multiple comparisons test all conditions were tested against the solvent control DMSO. * $p \leq 0.05$, ** $p \leq 0.01$, **** $p \leq 0.0001$, ns: $p>0.05$.

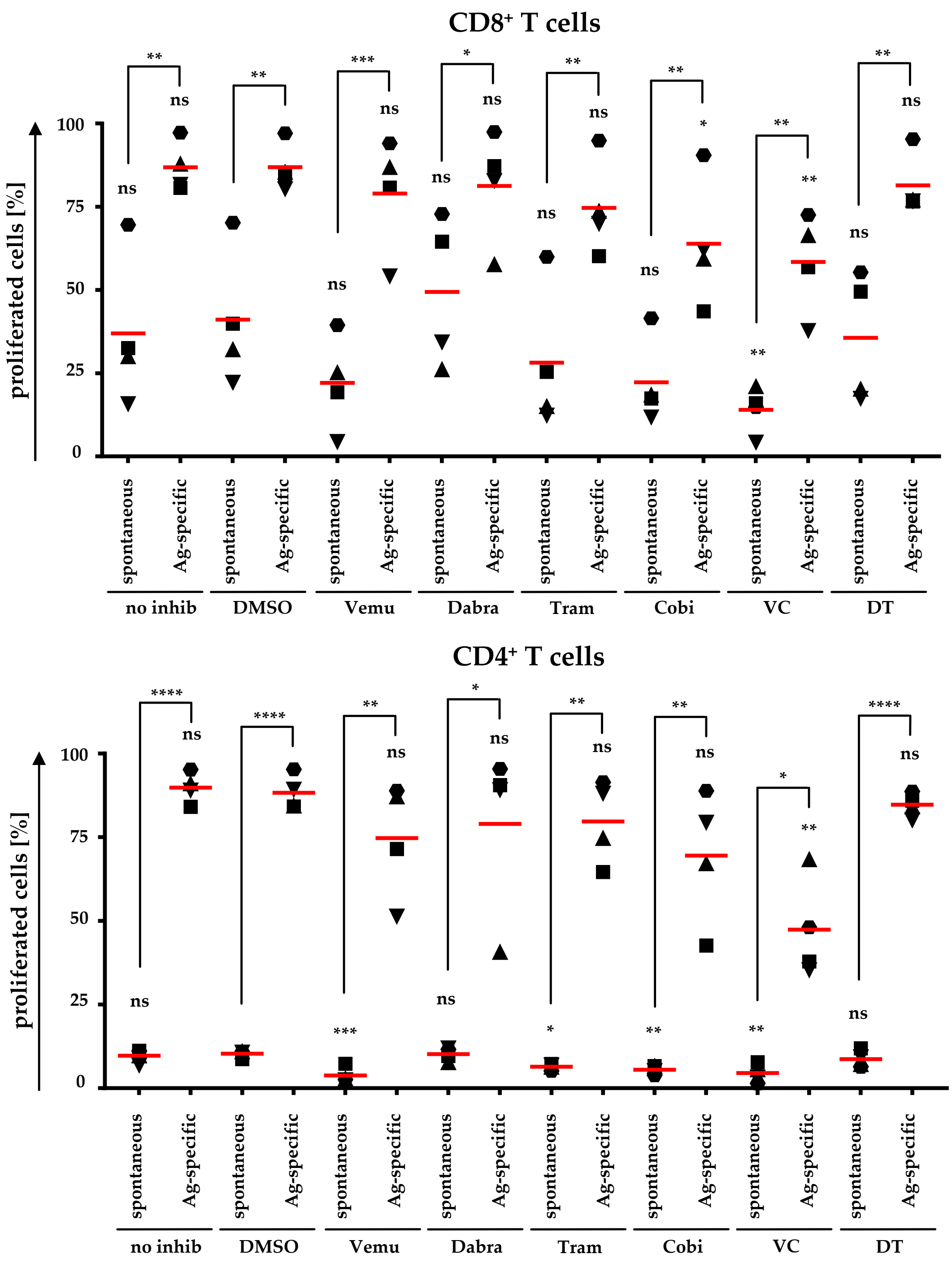

Figure S2. V+C-treatment reduces both unspecific and antigen-specific proliferation of CD8⁺ and especially CD4⁺ T cells upon DC-stimulation: moDCs were generated and matured as described above. DCs were additionally treated without inhibitor (no inhib), with solvent control (DMSO), vemurafenib (vemu), dabrafenib (dabra), trametinib (tram), cobimetinib (cobi), or the clinically used combinations VC or DT during the maturation process. After 24h hours, DCs were harvested either left untreated or were loaded with the respective gp100 peptide. CD4⁺ and CD8⁺ T cells were electroporated with RNA coding for the gp100-specific TCR and subsequently labelled with CFSE. CFSE-labelled T cells were co-incubated with gp100-loaded or non-loaded DCs at a 1:1 ratio in the presence of solvent control (DMSO), vemurafenib (vemu), dabrafenib (dabra), trametinib (tram), cobimetinib (cobi), or the clinically used combinations VC or DT, or without inhibitor treatment (no inhib). After three days, cells were harvested and analyzed by flow cytometry. To assess the antigen-specific vs. the spontaneous proliferation, we calculated the percentage of proliferated CD8⁺ or CD4⁺ T cells either after antigen-specific (gp100 peptide) or unspecific (no peptide) stimulation, respectively. Data of four donors (represented by different symbols) assessed

in independent experiments are shown. Red bars indicate mean values. p-values were determined by one-way ANOVA or paired student's t-test. In the Dunnett's multiple comparisons test all conditions were tested against the solvent control DMSO. To compare the spontaneous with the antigen-specific proliferation, p-values were assessed by paired-student's t-test for each inhibitor. * $p \leq 0.05$, ** $p \leq 0.01$, *** $p \leq 0.001$, **** $p \leq 0.0001$, ns: $p>0.05$.

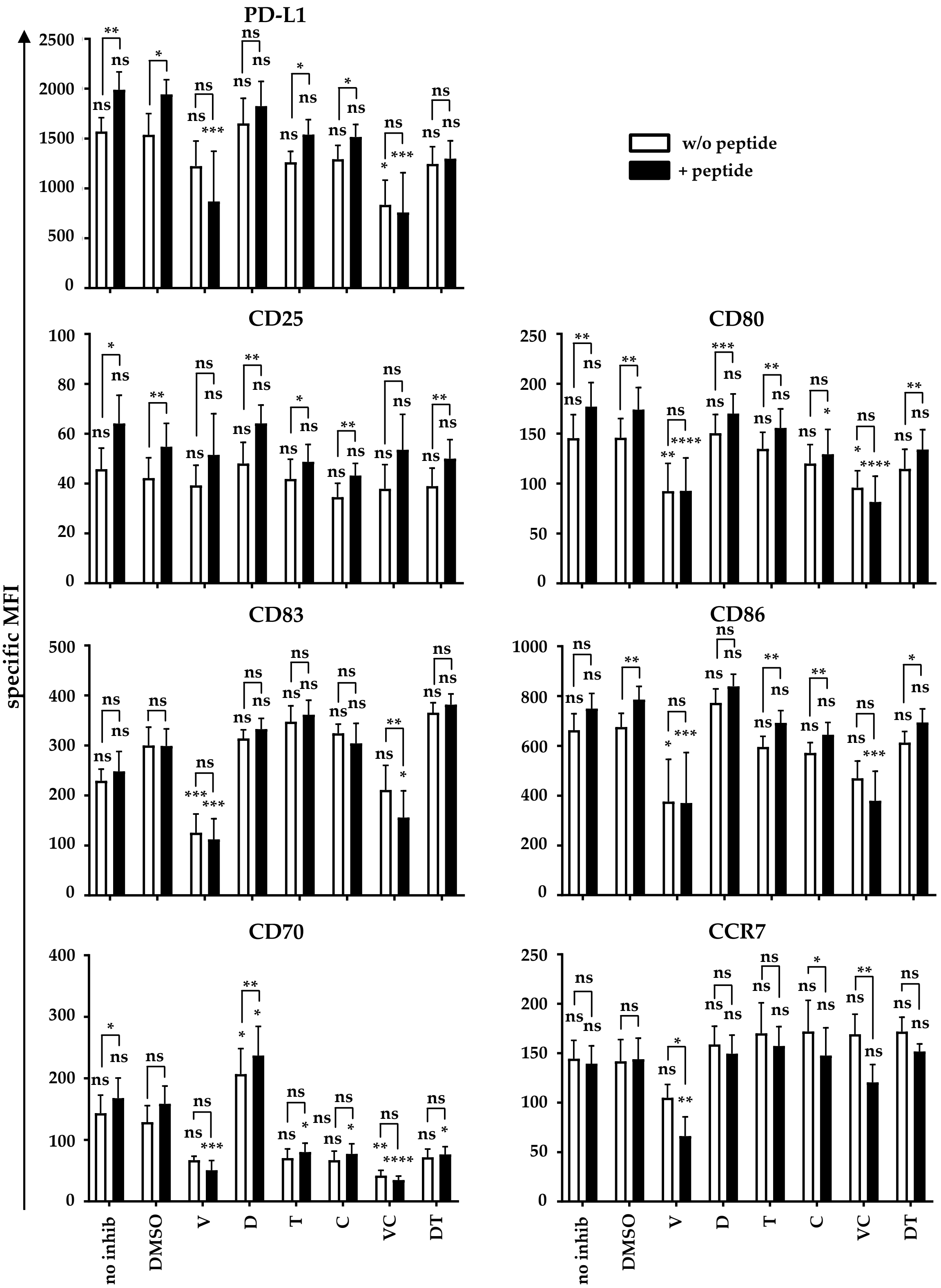

Figure S3. BRAF and MEK inhibitors do also affect the phenotype of moDCs after stimulation with CD8⁺ T cells: CD8⁺ T cells were transfected with a gp100-specific TCR. moDCs were generated as described before and were substituted during the maturation process with solvent control (DMSO), vemurafenib (V), dabrafenib (D), trametinib (T), cobimetinib (C), the clinically used combinations VC or DT, or without inhibitor (no inhib). After 24 h, DCs were either pulsed with the gp100 peptide (+ peptide, black bars) or were left untreated (w/o peptide, white bars). DCs and CD8⁺ T cells were co-cultured at a 1:1 ratio in the presence of solvent control (DMSO), vemurafenib (V), dabrafenib (D), trametinib (T), cobimetinib (C), the clinically used combinations VC or DT, or without inhibitor treatment (no inhib). After 24 hours, cells were harvested, stained for the indicated markers, and analyzed by flow

cytometry. The expression of surface markers on DCs is depicted as specific MFI (i.e. MFI after subtraction of background MFI of the respective isotype control antibodies). Data of six donors (represented by different symbols) assessed in independent experiments are indicated as mean values ± SEM. p-values were determined by one-way ANOVA or student's t-test. In the Dunnett's multiple comparisons test all conditions were tested against the solvent control DMSO for peptide and non-peptide conditions, respectively. To compare unspecific with antigen-specific surface marker expression, p-values were assessed by paired-student's t-test for each inhibitor. * $p \leq 0.05$, ** $p \leq 0.01$, *** $p \leq 0.001$, **** $p \leq 0.0001$, ns: $p>0.05$.
